# Relationship between *H. pylori* infection and some other risk factors in the incidence and recurrence of idiopathic nephrotic syndrome in children 

**DOI:** 10.22088/cjim.14.3.454

**Published:** 2023

**Authors:** Farrokh Farrokhnia, Ali Derakhshan, Mohammadhossein Fallahzade, Mitra Basiratnia

**Affiliations:** 1 Nephro-Urology Research Center, Shiraz University of Medical Sciences, Shiraz, Iran

**Keywords:** Infection, H. Pylori, Idiopathic nephrotic syndrome, Children

## Abstract

**Background:**

Idiopathic nephrotic syndrome (INS) is one of the chronic diseases in children and it is important to identify its related factors. The present study aimed at investigating the relationship between *H. pylori* infection and the incidence and recurrence of idiopathic nephrotic syndrome in children.

**Methods:**

The total number of case participants was 40 and the total number of control participants was 41. Based on the number of cases of nephrotic syndrome (NS), the same number of healthy children of the same age and gender were selected as the control group. The information and data collected include demographic characteristics of patients, duration of disease, number of recurrences, blood pressure and blood excretion in urine, height, and weight, and presence of gastrointestinal symptoms on the checklist. The data were entered into SPSS and analyzed at a significance level of 05.

**Results:**

From 81 participants in the study, 11 (13.75%) cases were *H. pylori* positive, of whom 7 (17.5%) cases were in the control group and 4 (10%) cases were in the patients’ group. There was no significant difference between the two groups in terms of *H. pylori* infection rate (P = 0.863). Moreover, there were no significant differences between the patients suffering from the nephrotic syndrome in terms of height, weight, blood pressure, hematuria, duration and recurrence of the disease (p>0.05).

**Conclusion:**

According to the evaluations performed in the present study, there was no relationship between low birth weight, blood pressure, disease duration, and disease recurrence with NS.

Idiopathic nephrotic syndrome (INS) is one of the most important and chronic kidney diseases in children and its prevalence in different populations has been reported to range from 1.15 to 16.9 per 100000 people (1). Nephrotic syndrome (NS) can be congenital or acquired; the acquired type can be associated with systemic diseases, genetic disorders, and primary (idiopathic) or secondary infections (2, 3). The main form of this disease is mostly childhood NS. The treatment of acute NS in children has commonly started without any need for a kidney biopsy. The treatment period is long and sometimes associated with frequent recurrences (4).

Various studies have reported the prevalence of recurrence to be between 60-80%; this recurrence results in treatment problems and parents’ concerns on the prognosis and future of their children (2-4). Various factors have been known to be effective in the recurrence of NS, the most important of which are viral infections. Determining the factors associated with the development of the disease, the ability to predict the course of the disease, and the prognosis of patients suffering from nephrotic syndrome have significant effects on adopting a correct treatment process and reducing parental concerns; recent studies have, thus, focused on identifying the risk factors.

There is evidence indicating that bacteria are involved in the development of nephropathy (5, 6). The previous studies have shown a relationship between Proteinuria and *H. pylori* infection (7). Some other studies have also indicated that infection *H. pylori* intensifies renal function and causes further deposition of antigen A nephropathy (8-10). Therefore, even if *H. pylori* infection is not directly involved in the development of nephrotic syndrome, the rate of proteinuria in this disease may be reduced by treating the affected individuals. Thus, finding a link between *H. pylori* infection and NS is important and there is hope to improve the treatment of the disease by eliminating the secondary factor.

*Helicobacter pylori* is more common in developing countries; it has involved almost half of the world, with most cases occurring in developing countries (11). In most cases, *H. pylori* infection occurs early in life in children younger than 5 years old, and the person becomes chronically infected (12). The most important risk factors for infection include socioeconomic status, busy/noisy living environment, and lack of hygiene (13). Evidence that bacteria are involved in causing nephropathy is increasing. Some studies show that *H. pylori* infection affects the production and glycosylation of IgA1, intensifying renal function and causing more severe levels of antigen deposition in IgA nephropathy (14, 15). According to this content, the present study aimed at investigating the relationship between *H. pylori* infection and acute INS and its recurrence, so that the cause of development and required interventions will be more accurately investigated. In addition to investigating this relationship, the present study has also attempted to investigate the relationship between low weight, hematuria, blood pressure, duration of disease, and its recurrence in patients with nephrotic syndrom.

## Methods

The present study is a case-control study, all new cases of children aged 1 to 12 years with a diagnosis of the INS as well as children diagnosed with a previous NS with a recurrence of the disease (after confirmation of the diagnosis by a pediatric nephrologist) from September 23rd, 2020 to March 20th, 2021 referred to the gastrointestinal and liver center of Namazi Hospital (Tehran, Iran). The cases who entered the present study were those who had been introduced to perform *H. pylori* antigen on fecal samples. Children who entered the present study were those with NS who had stopped taking their drugs for more than two months and were “in remission” and experienced the recurrence of the disease. 

Exclusion criteria included congenital nephrotic syndrome, secondary nephrotic syndrome due to immune disorders, specific infectious diseases and medications, renal failure and hypertension, taking antibiotics and antacids, and unwillingness to participate in the study. The total number of case group participants was 40 and the total number of control group participants was 40. In the present study, group matching was performed. Based on the number of cases of NS, the same number of healthy children of the same age and gender referred to the gastrointestinal and liver center of Namazi Hospital (Tehran, Iran) were selected as the control group. 

**Method of conducting the experiments: **Stool antigen was conducted by enzyme immunoassay using a special kit (Meridian Diagnostics Inc., Cincinnati, OH) on a fresh stool sample. If fecal antigen was positive for *H. pylori*, endoscopy and biopsy were conducted by a gastroenterologist, and samples were sent for rapid urease and histological examination. Urea membrane kit (pylori tek, serim research, Elkhart, IN, and helicocheck, Institute of Immunology, Co., Ltd., Tochigi, Japan) was used to conduct a rapid urease test. A gastric biopsy specimen is placed between a reagent strip with a pH indicator and a pad containing urea, and within a maximum of 4 hours, discoloration indicates *H. pylori* infection. 

For histological examination, the gastric biopsy specimen was examined for *H. pylori* by a pathologist after special Giemsa staining. Total rapid urease test and histological examination were considered as the gold standard for *H. pylori* diagnosis. Since there are different methods such as endoscopy, respiratory test, blood test, antibody level measurement, fecal antigen test for proving the infection, it was primarily attempted to use the most non-invasive method (fecal antigen test for *H. pylori*). In addition to being non-invasive, the sensitivity and specificity of this test are 98% and 100%, respectively (16).

**Data collection tools and procedure: **The information and data include demographic characteristics of patients, duration of disease, number of recurrences, blood pressure and blood excretion in urine, height, and weight, and the presence of gastrointestinal symptoms on the checklist. Informed consent was obtained from parents of children whose children had entered the study as case and control participants after providing the required explanations.

**Data analysis: **The results were expressed as mean and standard deviation (mean ± SD) for quantitative variables and as a percentage for qualitative variables. The normality of data distribution was evaluated by the Kolmogorov-Smirnov test. The chi-square test was used for the analysis of recurrence of the disease and the frequency of sex of participations. Student t-test was performed to compare the mean age, height, weight, BMI, systolic BP (mmHg), and diastolic BP (mmHg). The Fisher's exact test was used to evaluate the frequency of recurrence. A significant level was considered less than 0.05. SPSS software Version 23 was used for the statistical analysis of data.

## Results

In the control group, the minimum age was 1 year and the maximum was 12 years, and the mean age was 5.4 years, and the case group had a mean age of 5.9 years. From 40 patients in the case group, as many as 13 (32.5%) were new cases and 27 (67.5%) cases were the known ones under treatment. Of the 80 participants in the present study, 11 (13.75%) cases were *H. pylori* positive, of whom 7 (17.5%) were in the control group and 4 (10%) cases were in the case group. There was no significant difference between the two groups in terms of *H. pylori* infection (P=0.863) (table 1). Endoscopy was conducted (after obtaining their consent) for two of the 4 patients who were confirmed to be positive for *H. pylori*. The same findings were confirmed for *H. pylori* infection. None of the cases showed clinical and endoscopic findings. In the case group, 7 (17.5%) of 40 patients had hematuria. In terms of blood pressure, none of them had high blood pressure. The height distribution of patients ranged from 82 to 146 cm, the average of whom was about 112 cm. The weight distribution of patients ranged from 10 to 42 kg with an average of 21.7 kg. There was no statistically significant relationship between height, low birth weight, BMI, blood pressure of participants and NS (p>0.05) (table 2). 

The results of investigating the patients in terms of duration of the disease and dividing the patients into three-time groups indicated that as many as 13 (32.5%) patients were at the onset of the disease (new cases), 2 patients (5%) patients had passed less than one year of their disease, and 25 (62%) patients had passed more than a year since the onset of their disease. As for the number of recurrences, 21 (52.5%) patients had no history, 5 (12.5%) patients had experienced 1 recurrence, 5 (12.5%) patients had experienced two recurrences, and the remaining 9 (12.5%) patients had experienced three or more recurrences of the disease. There was no significant relationship between the duration and recurrence of NS with *H. pylori* infection (p>0.05) (table 3). 

**Table 1:** Frequency, distribution and percentage of H. pylori infection in control and experimental groups

**Groups being investigated**	***H. pylori*** ** positive**	***H. pylori*** ** negative**	**Frequency (percentage)**	**Significance level**
**Control**	7 (17.5%)	34 (82.5%)	40 (100%)	0.863
**Case**	4 (10%)	36 (90%)	40 (100%)	

**Table 2:** Mean and standard deviation of height, weight and BMI in experimental and control group

**Variable**	**Group**	**Mean ± SD** **or percentage**	**P-value**
**Height (cm)**	Case	112± 18.5	0.067
	Control	111±18	
**Weight (kg)**	Case	21.7 ±7.6	0.217
	Control	22±7.4	
**BMI**	Case	17±2.8	0.084
	Control	16±2.7	
**Systolic BP (mmHg)**	Case	119±22	0.865
	Control	133±18	
**Diastolic BP (mmHg)**	Case	76±13	0.540
	Control	78±10	

BMI: Body Mass Index

BP: Blood Pressure

**Table 3:** Frequency distribution, percentage of duration and recurrence of the disease

**Variable**	**Status**	**Case**		**Control**		**P-value**
		**Frequency**	**Percentage**	**Frequency**	**Percentage**	
**Duration of the disease**	New case	13	32.5	14	34.1	0.657
	Less than 1 year	2	5	4	0.09	
	More than 1 year	25	62.5	23	0.56	
	Total	40	100	41	100	
**Disease recurrence**	Without any recurrences	21	52.5	20	48.7	0.314
	1 recurrence	5	12.5	6	14.3	
	2 recurrences	5	12.5	6	14.3	
	3 and more recurrences	9	22.5	7	17	
	Total	40	100	41	100	

## Discussion

The global prevalence of *H. pylori* infection has been reported to vary from 10% to 80% for different ages (11). The available statistics show that the rate is about 10% in Western countries, while in developing countries including Iran, the rate has been reported to be up to 90% for some specific ages of children (11, 17). The percentage of children with *H. pylori* infection in the present study is 13.6%, somewhat different from other studies' findings. The prevalence of this infection in the study conducted by Kienesberger et al. (2018) was respectively 8.9%, 36.4%, and 31.9% for children aged one, two, and four years (18). Moreover, in the study conducted by Zhou et al. (19) (2018), the rate was reported to be 24.1, and in the study conducted by Abbas et al. (20) (2018), it was 21.8%. Perhaps the reason behind the different rates of the present study is the type of study and the method of selecting the children of the control group (from the affluent urban class), or the small number of participants in each group.

Statistical comparison between the control and case groups in the present study did not show a significant difference in terms of *H. pylori* infection (17.5% vs. 10%). Thus, it can be concluded that *H. pylori* infection may not be associated with NS. This finding is in line with the results of the study conducted by Liu et al. (2020) (21). However, it is not in line with the findings of other studies that have shown that microbial infection, and in particular *H. pylori* infection, is associated with NS (7-10). Perhaps one of the causes of *H. pylori* infection, according to other studies (8, 22), is high doses of steroids, which make patients more susceptible to *H. pylori* infection. Previous studies have indicated that treatment of *H. pylori* infection reduces proteinuria (3, 23), and whether the source of the NS is *H. pylori* infection or not, treatment of this infection seems to be necessary for patients.

Numerous factors of weight and height of sick children (BMI) can affect the development of NS in children including diet, side effects of medications, the presence of an underlying disease, duration of the disease, and infections. Low birth weight is associated with increased cardiovascular diseases, diabetes, hypertension, and morbidity throughout life. In this study, according to the average age of the experimental group, height and weight criteria were in the normal range. Thus, it can be stated that this finding is not in line with that of the previous studies; low birth weight is not associated with the NS. Previous studies have indicated that reduced nephrons in neonates result in serious damage to kidneys by increasing the size of the glomerulus and the hyperfiltration arising from that (24-26).

In the present study, no significant relationship was found between the duration of disease, and recurrence of NS in the children and *H. pylori* infection. Hematuria was reported for 17.5% of the patients; this is in line with the results of the study conducted by Avner et al. with about 20% (27). None of the sick children in this study had high and uncontrolled hypertension. This is not in line with the results of the studies conducted by Xing-Zi Liu et al. (20) (2020) and Kanbay et al. (28) (2007); they have indicated that blood pressure in patients with *H. pylori* infection was higher. The reason behind this discrepancy could be the different sample sizes and ages of the participants in this study; the present study was conducted on children, but the average age of participants in the study conducted by Liu et al. was 37.7 years. The limitation of our study was due to the fact that cases of children with NS are rare, collecting large numbers of patients at the time of the project was not possible, and the sample size of this study was collected based on existing limitations. The finding of the present study showed that there was no significant relationship between *H. pylori* infection, low birth weight, and blood pressure of children and incidence and recurrence of INS.
